# Informal Caregiving, Chronic Physical Conditions, and Physical Multimorbidity in 48 Low- and Middle-Income Countries

**DOI:** 10.1093/gerona/glaa017

**Published:** 2020-01-16

**Authors:** Louis Jacob, Hans Oh, Jae Il Shin, Josep Maria Haro, Davy Vancampfort, Brendon Stubbs, Sarah E Jackson, Lee Smith, Ai Koyanagi

**Affiliations:** 1 Faculty of Medicine, University of Versailles Saint-Quentin-en-Yvelines, Montigny-le-Bretonneux, France; 2 Research and Development Unit, Parc Sanitari Sant Joan de Déu, CIBERSAM, Sant Boi de Llobregat, Barcelona, Spain; 3 University of Southern California, Suzanne Dworak Peck School of Social Work, Los Angeles; 4 Department of Pediatrics, Yonsei University College of Medicine, Seoul, Republic of Korea; 5 Department of Rehabilitation Sciences, KU Leuven, Belgium; 6 University Psychiatric Center, KU Leuven, Belgium; 7 Institute of Psychiatry, Psychology and Neuroscience, King’s College London, UK; 8 South London and Maudsley NHS Foundation Trust, Denmark Hill, UK; 9 Department of Behavioural Science and Health, University College London, UK; 10 The Cambridge Centre for Sport and Exercise Sciences, Anglia Ruskin University, Cambridge, UK; 11 ICREA, Barcelona, Spain

**Keywords:** Informal caregiving, Chronic physical conditions, Physical multimorbidity, Low- and middle-income countries, Cross-sectional multicountry study

## Abstract

**Background:**

The health of the caregivers is crucial to sustain informal care provision, while multimorbidity is an important health risk concept. However, studies on the association between informal caregiving and physical multimorbidity are currently lacking. Therefore, we investigated this association in adults from 48 low- and middle-income countries (LMICs).

**Method:**

Cross-sectional data from 242,952 adults (mean age 38.4 years) participating in the World Health Survey 2002–2004 were analyzed. Informal caregivers were considered those who provided help in the past year to a relative or friend (adult or child) who has a long-term physical or mental illness or disability, or is getting old and weak. Nine physical conditions were assessed. Multivariable logistic regression analyses were conducted to assess associations between informal caregiving and physical multimorbidity, while the between-country heterogeneity in this relationship was studied with country-wise analyses.

**Results:**

The overall prevalence of informal caregiving and physical multimorbidity (ie, two or more physical conditions) was 19.2% and 13.2%, respectively. Overall, caregivers had 1.40 (95% confidence interval = 1.29–1.52) times higher odds for physical multimorbidity. This association was particularly pronounced in younger caregivers (eg, 18–44 years: odds ratio = 1.54; 95% confidence interval = 1.37–1.72), whereas this association was not statistically significant among those aged ≥65 and older (odds ratio = 1.19; 95% confidence interval = 0.98–1.44). Country-wise analyses corroborated these findings, and there was a negligible level of between-country heterogeneity (*I*^2^ = 24.0%).

**Conclusions:**

In LMICs, informal caregivers (especially young caregivers) were more likely to have physical multimorbidity. This should be taken into account in policies that address the health and well-being of informal caregivers.

Population aging is a major challenge for modern societies (1). The global proportion of people aged over 60 years is estimated to increase from 12% in 2015% to 22% in 2050, with 80% of the older adults living in low- and middle-income countries (LMICs) (2). One consequence of population aging is an increasing number of individuals in need of care (3,4). The vast majority of these individuals are provided unpaid care from relatives or friends (informal care) (5). Informal care is an important alternative to expensive health care services and institutional care. In LMICs, access to health and welfare service is limited, and there is a particularly heavy reliance on informal caregivers (6). Thus, the health of the caregivers is crucial to sustain informal care provision, especially in LMICs.

Despite this, there are only a few studies on the health status of caregivers in LMICs. These studies have shown that caregivers in LMICs are more likely to have mental or physical health problems (7), but there are currently no studies on the association between physical multimorbidity (ie, two or more chronic physical conditions) and caregiving. Studies on multimorbidity are crucial as it is an important risk concept associated with increased disability (8,9), poorer quality of life (10), and premature mortality (11). Caregivers are known to be at increased risk of stress (12), sleep problems (13), and unhealthy behavior such as smoking (14) and lack of physical activity (15), and these may increase risk for multimorbidity. Moreover, one previous U.S. study including 359 spousal caregivers and care recipients found that caregivers with multiple chronic conditions had greater emotional and physical difficulties than those without multiple chronic conditions (16), underlying the point that the presence of multiple chronic conditions may favor the onset of negative care-related outcomes (eg, poor physical health, subjective burden, low care-related quality of life). Reverse causality is also possible, and multimorbidity may be a significant predictor of informal caregiving. For example, young individuals with multimorbidity are less likely to be employed (17), and thus may have more time at home to provide informal caregiving for relatives.

Therefore, the main goal of this study was to investigate associations of informal caregiving with nine chronic physical conditions (ie, angina, arthritis, asthma, chronic back pain, diabetes, edentulism, hearing problem, tuberculosis, visual impairment) and physical multimorbidity in adults from 48 LMICs. We also assessed whether associations differ by sex and age. In particular, working-age adults often provide financial support to their older relatives in LMICs, and we hypothesized that this may be an additional stressor and may indirectly increase the risk for chronic physical conditions especially in this age group (18).

## Method

### The Survey

The World Health Survey (WHS) was a cross-sectional, community-based study undertaken in 2002–2004 in 70 countries worldwide. Details of the survey are provided in the World Health Organization (WHO) website (http://www.who.int/healthinfo/survey/en/). Briefly, data were collected using stratified multistage random cluster sampling. Individuals aged 18 and older with a valid home address were eligible to participate. Each member of the household had an equal probability of being selected by utilizing Kish tables. A standardized questionnaire, translated accordingly, was used across all countries. The individual response rate across all countries was 98.5% (19). Ethical approval to conduct the study was obtained from the ethical boards at each study site. Informed consent was obtained from all participants. Sampling weights were generated to adjust for nonresponse and the population distribution reported by the United Nations Statistical Division.

Data were publicly available for 69 countries. Of these, 10 countries were excluded due to a lack of sampling information. Furthermore, 10 high-income countries were excluded to focus on LMICs. Moreover, Turkey was deleted due to lack of data on caregiving. Thus, the final sample consisted of 48 LMICs (*n* = 242,952) according to the World Bank classification at the time of the survey (2003). The list of the included countries and their sample size are provided in Supplementary Table S1 of Appendix. The data were nationally representative for all countries with the exception of China, Comoros, India, Ivory Coast, the Republic of Congo, and Russia.

### Physical Health Conditions

A total of nine physical conditions were assessed (ie, angina, arthritis, asthma, chronic back pain, diabetes, edentulism, hearing problem, tuberculosis, visual impairment). We included all physical conditions available in the WHS. Angina was assessed using a self-reported diagnosis and a symptom-based diagnosis based on the Rose questionnaire. Arthritis, asthma, and diabetes mellitus were based on self-reported lifetime diagnosis. Chronic back pain was defined as having had back pain (including disc problems) everyday during the last 30 days. Edentulism was assessed by the question “Have you lost all your natural teeth?” Those who responded affirmatively were considered to have edentulism. The participant was considered to have hearing problems if the interviewer observed that the participant had difficulty hearing throughout the survey. A tuberculosis diagnosis was based on past 12-month symptoms and was defined as (a) having had a cough that lasted for 3 weeks or longer and (b) having had blood in phlegm or coughed up blood. Finally, visual impairment was defined as having extreme difficulty in seeing and recognizing a person that the participant knows across the road (ie, from a distance of about 20 m). A validation study showed that this response probably corresponds to WHO definitions of visual impairment (20/60 or 0.48 log-MAR) (20). We calculated the total number of these conditions while allowing for one missing variable in order to retain a larger sample size. Physical multimorbidity was defined as having at least two conditions, in line with previously used definitions (4).

### Informal Caregiving

Those who answered affirmatively to the question “During the past year, did you provide help to a relative or friend (adult or child), because this person has a long-term physical or mental illness or disability, or is getting old and weak?” were considered to be informal caregivers. This question is comparable to those used in previous surveys to identify caregivers where participants were asked whether or not they look after, or give help or support to family members, friends, neighbors, or others because they have a long-term physical or mental ill-health or disability, or problems related to age (21).

### Control Variables

Control variables were chosen based on past literature (22) and included age, sex, highest education achieved (primary or less/secondary or higher), country-wise wealth quintiles, and employment status (employed or not employed). The wealth quintiles were created using principal component analysis based on 15–20 assets including country-specific items for some countries.

### Statistical Analysis

Statistical analyses were performed with Stata 14.1 (Stata Corp LP, College Station, TX). The difference in sample characteristics between those who do and do not provide care was analyzed using chi-squared tests. Multivariable logistic regression analysis was conducted to assess the association between informal caregiving (exposure) and physical multimorbidity (outcome), adjusting for covariates. Analyses using the overall sample and samples stratified by age (18–44, 45–64, ≥65 years), and sex were conducted. Interaction analysis was conducted to assess whether the difference in magnitude of the association between age and sex groups is statistically significant by including product terms of age × caregiving and sex group × caregiving in the models. To assess whether the association between informal caregiving and physical multimorbidity is consistent across countries, we conducted country-wise logistic regression analyses. The estimates for each country were also combined into a fixed-effect meta-analysis with the Higgins’s *I*^2^ statistic being calculated. This represents the degree of heterogeneity that is not explained by sampling error with values of 25%, 50%, and 75% often being considered as low, moderate, and high levels of heterogeneity, respectively (23).

All regression models were adjusted for age, sex, education, wealth, employment status, and country with the exception of the country-wise and sex-wise analyses, which were not adjusted for country and sex, respectively. Adjustment for country was conducted by including dummy variables for each country. All variables were used in the regression analysis as categorical variables with the exception of age (continuous variable). Taylor linearization methods were used in all analyses to account for the sample weighting and complex study design. Results from the logistic regression analyses are presented as odds ratios (ORs) with 95% confidence intervals (CIs). The level of statistical significance was set at *p* < .05.

## Results

**Figure 1.**
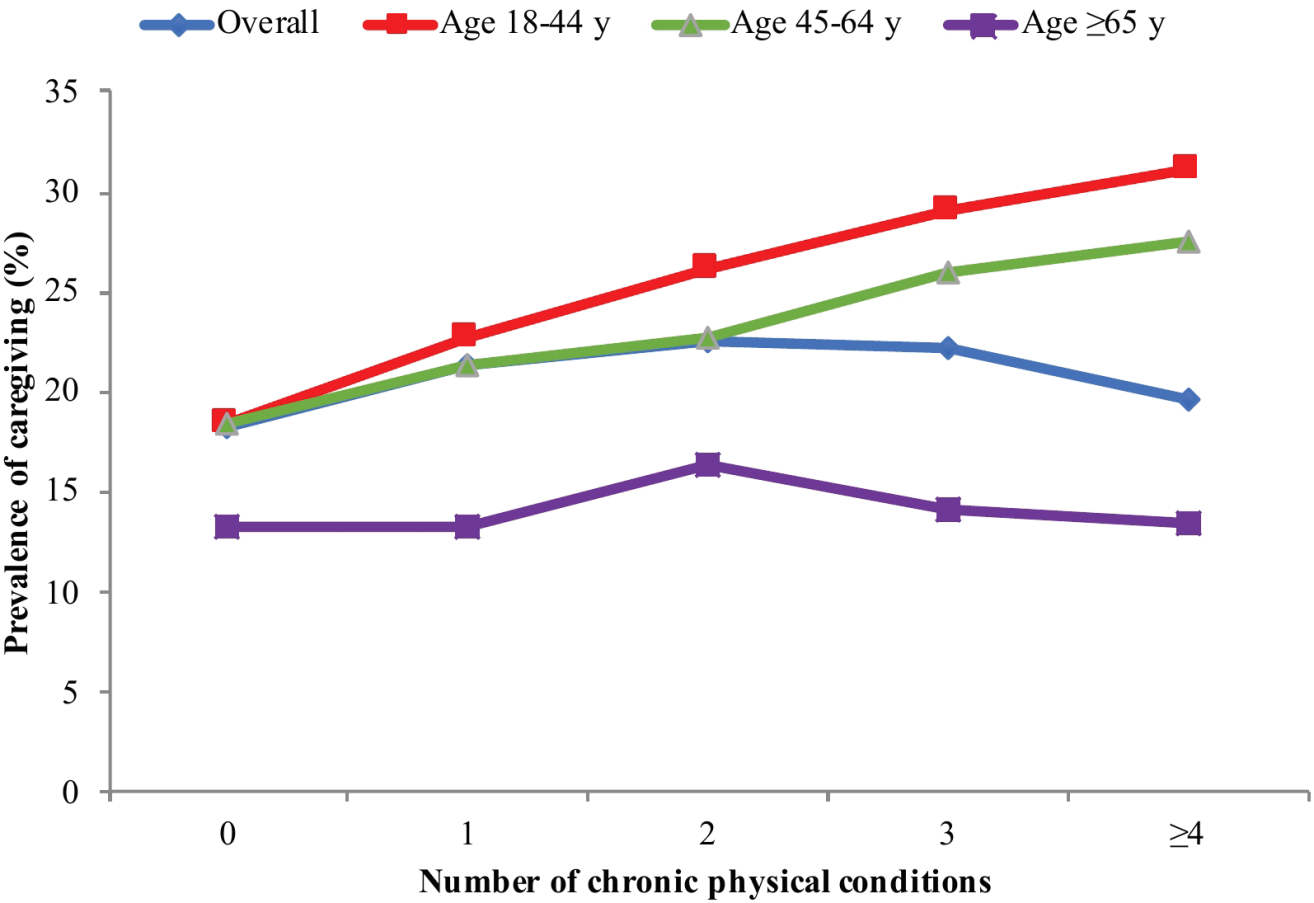
Prevalence of caregiving by number of chronic physical conditions.

**Figure 2.**
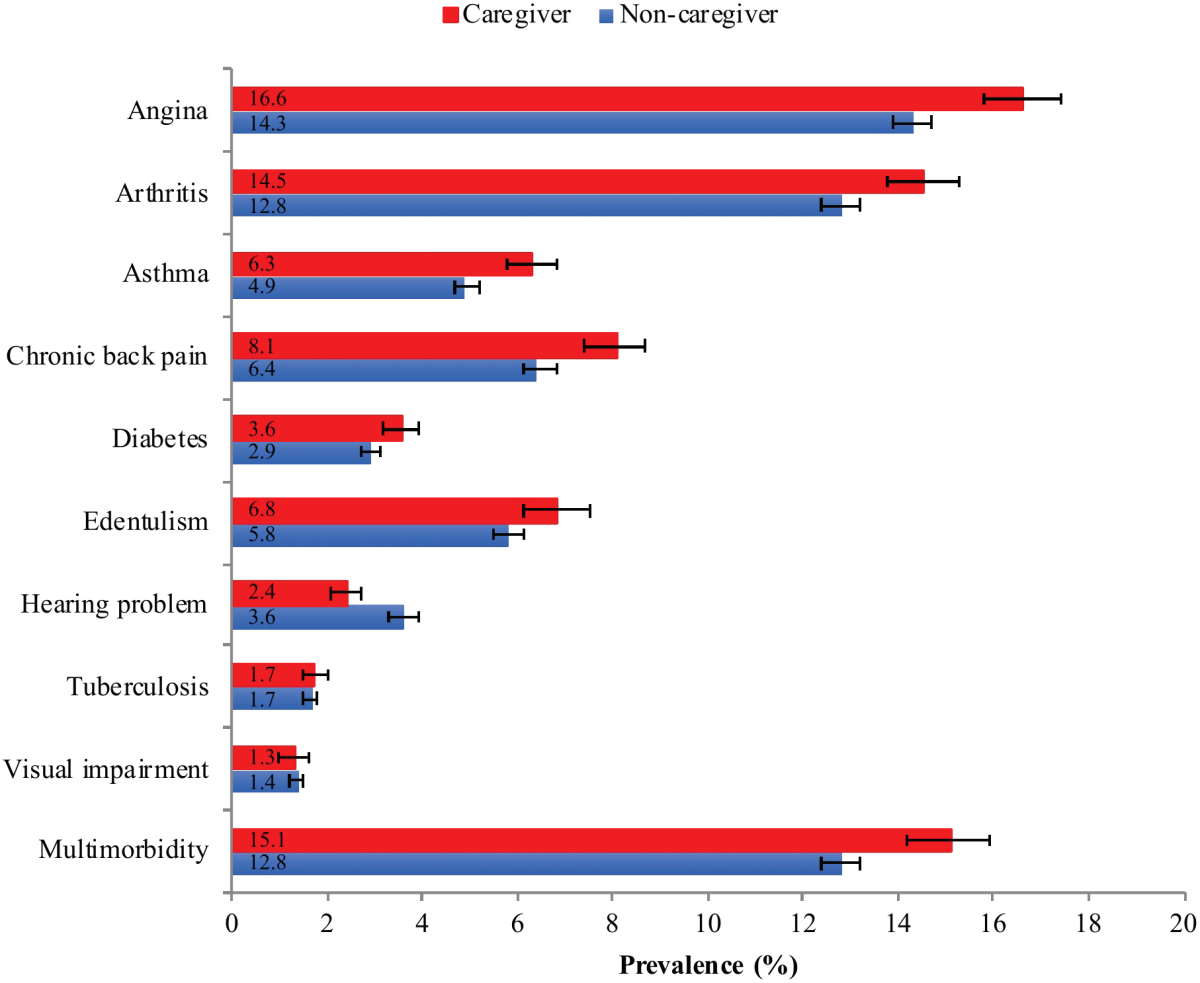
Prevalence of chronic physical conditions and multimorbidity among noncaregivers and caregivers. Bars denote 95% confidence intervals. Multimorbidity was defined as two or more chronic conditions.

**Figure 3.**
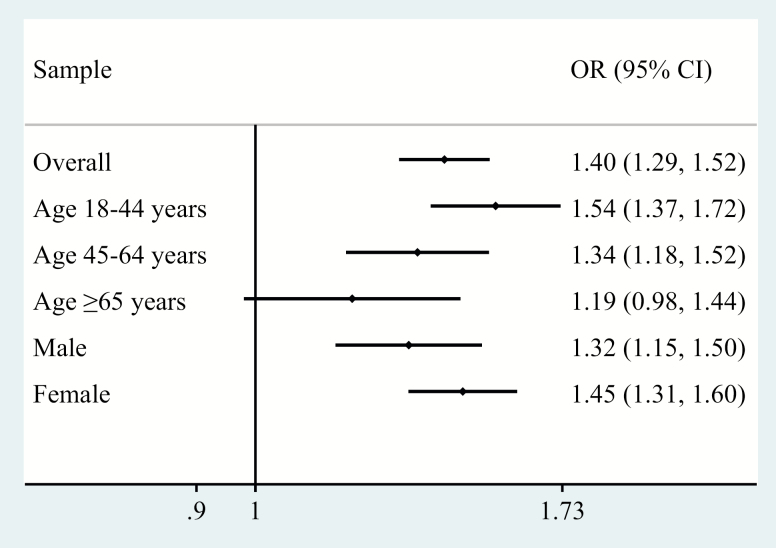
Association between caregiving (exposure) and multimorbidity (outcome) estimated by multivariable logistic regression. Models are adjusted for age, sex, education, wealth, employment, and country with the exception of samples consisting only of males or females, which were not adjusted for sex. Multimorbidity was defined as two or more chronic conditions. OR = odds ratio; CI = confidence interval.

**Figure 4.**
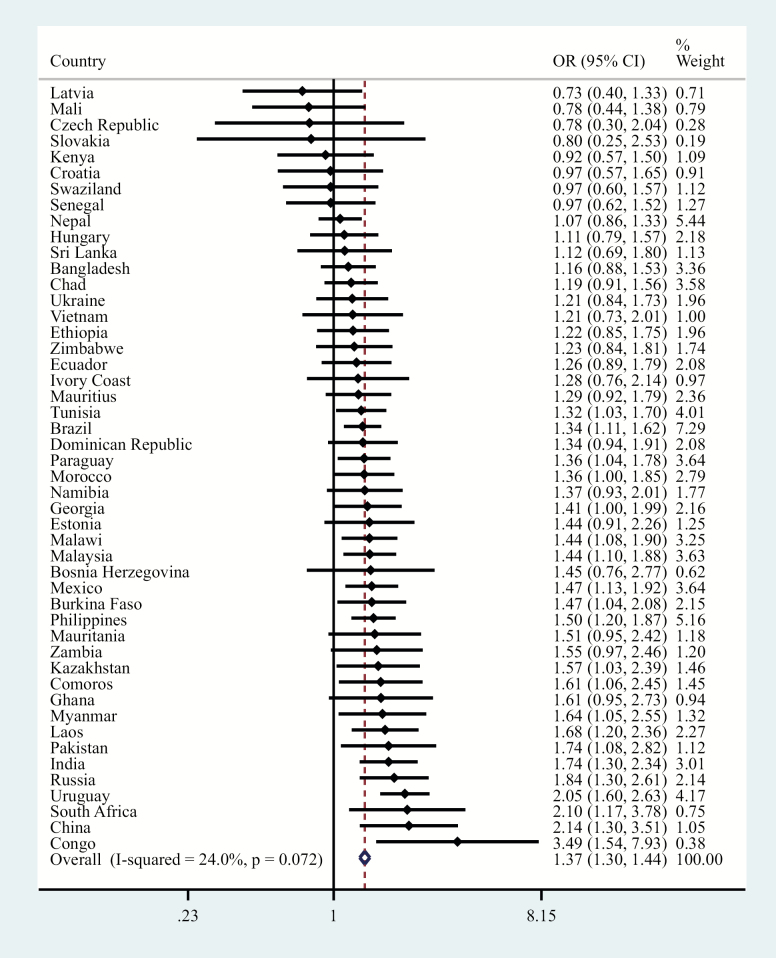
Country-wise association between caregiving (exposure) and multimorbidity (outcome) estimated by multivariable logistic regression. Models are adjusted for age, sex, education, wealth, and employment. Overall estimate was obtained by meta-analysis with fixed effects. Multimorbidity was defined as two or more chronic conditions. OR = odds ratio; CI = confidence interval.

The final sample consisted of 242,952 adults aged ≥18 and older (mean [*SD*] age 38.4 [16.0] years; 50.8% female). The overall prevalence of informal caregiving and physical multimorbidity was 19.2% and 13.2%, respectively. The sample characteristics are provided in Table 1. Caregivers were more likely to be younger, females, and have higher education and greater levels of wealth, whereas they were also less likely to be unemployed. The prevalence of informal caregiving by number of chronic physical conditions is shown in Figure 1. There was a linear increase in the prevalence of informal caregiving with increasing number of chronic physical conditions among those aged ≤64 years. However, this trend was not observed among those aged ≥65 and older, with the prevalence of caregiving decreasing beyond two chronic physical conditions. The prevalence of chronic physical conditions and physical multimorbidity among caregivers and noncaregivers are shown in Figure 2. Caregivers were significantly more likely than noncaregivers to have angina, arthritis, asthma, chronic back pain, diabetes, edentulism, and multimorbidity, whereas they were significantly less likely to have hearing problems. The association between informal caregiving and physical multimorbidity estimated by multivariable logistic regression is shown in Figure 3. Overall, caregivers had 1.40 (95% CI = 1.29–1.52) times higher odds for physical multimorbidity. The difference in the strength of the association between the youngest and the oldest age group was statistically significant (ie, significant interaction), whereas there was no evidence of interaction by sex. Specifically, the OR (95% CI) for those aged 18–44 years was 1.54 (1.37–1.72), but this did not reach statistical significance among those aged ≥65 and older (OR = 1.19; 95% CI = 0.98–1.44). Country-wise analyses showed a positive association between informal caregiving and physical multimorbidity in the majority of the countries. The overall OR (95% CI) based on a meta-analysis was 1.37 (1.30–1.44) with a negligible level of between-country heterogeneity (*I*^2^ = 24.0%; Figure 4).

**Table 1.** Sample Characteristics (Overall and by Caregiving Status)

Characteristic	Category	Overall	Caregiving		*p* Value
			No	Yes	
Age (y)	18–44	67.8	67.6	68.9	<.001
	45–64	23.6	23.2	24.9	
	≥65	8.6	9.2	6.2	
Sex	Male	49.2	49.7	47.3	<.001
	Female	50.8	50.3	52.7	
Education	<Secondary	57.3	58.7	50.8	<.001
	≥Secondary	42.7	41.3	49.2	
Wealth	Poorest	20.1	20.9	17.1	<.001
	Poorer	20.0	20.3	18.2	
	Middle	19.9	19.9	19.7	
	Richer	20.0	19.8	20.9	
	Richest	20.0	19.0	24.1	
Employment status	Employed	57.2	56.8	58.8	.001
	Unemployed	42.8	43.2	41.2	

*Notes*: Data are column percentages. *p* Value was calculated by chi-squared tests.

## Discussion

### Main Findings

In this multicountry study including almost 243,000 adults from 48 LMICs, we found that the prevalence of informal caregiving and physical multimorbidity was around 19% and 13%, respectively. In terms of the individual chronic conditions, caregivers were more likely to have angina, arthritis, asthma, chronic back pain, diabetes, and edentulism, but less likely to have hearing problems. After adjusting for several potential confounders, there was a significant, positive association between informal caregiving and physical multimorbidity (OR = 1.40), and this relationship was stronger in younger participants. These findings were corroborated in the country-wise analysis: informal caregiving was significantly associated with physical multimorbidity in the majority of countries with a low level of between-country heterogeneity. To the best of our knowledge, this is the first study investigating the relationship between informal caregiving and physical multimorbidity.

### Interpretation of the Findings

Although the causal pathways are still unclear, there are several potential explanations for the positive association between informal caregiving and chronic physical conditions or physical multimorbidity. First, informal caregiving may be a risk factor for these conditions. For example, previous research has found that informal caregivers are at a particular high risk for musculoskeletal discomfort and injury, especially when engaging in activities that are physically demanding (eg, transfers, bathing) (24). These activities may also increase risk for injury and possibly osteoarthritis (25) and lead to multiple chronic conditions. Furthermore, several longitudinal studies from high-income countries have found that caregiving is associated with an increased risk for future onset of cardio-metabolic diseases (26).

Moreover, the effects of informal caregiving on chronic physical conditions or physical multimorbidity may be mediated by several factors such as stress (12), sleep disturbance (13), depression (7), unhealthy behaviors (14,15), and lack of economic resources (27). For example, informal caregiving was a significant risk factor for perceived stress, sleep problems, and depression in a study of 258,793 adults from 58 countries (7), and these conditions are known risk factors for a variety of chronic physical conditions including cardiovascular diseases (28–30) and asthma (31). With respect to sleep, studies have shown that the adverse effects of sleep problems on cardiovascular health probably involve low-grade inflammation, increased cortisol secretion, and changes in circulating levels of leptin and ghrelin (30). In terms of unhealthy behavior, previous studies have shown that caregivers are more likely to smoke (14) and that the prevalence of problematic drinking patterns and sedentary behavior is high in this population (32). Caregivers may be more likely to smoke and drink alcohol to cope with the stress associated with caregiving, whereas caregivers may also be more prone to be sedentary for being at home more often to take care of their older or disabled relatives. Finally, previous research has found a positive association between earlier-life caregiving and later-life poverty (27), while low income is a risk factor for a numerous chronic physical conditions (eg, obesity, diabetes). It should be noted that the aforementioned mediators (ie, stress, sleep disturbance, unhealthy behavior, lack of economic resources) are interconnected and can work jointly to increase risk for physical multimorbidity.

It is also possible that caregivers neglect their own health as their health problems may seem less important compared with that of the care recipient. Moreover, caregivers may not have enough time or energy to attend health visits owing to the high caregiving burden and stress. For example, a study including 315 caregivers from the United States showed that the number of visits to the doctor was lower in caregivers than in the general population (between 45 and 64 years: 4.2 vs. 6.6; between 65 and 74 years: 5.6 vs. 8.1; and after 74 years: 5.7 vs. 10.6) (33). Another study conducted in Pakistan revealed that around 61% of informal caregivers neglect their health during caregiving (34). The cost of health care for the care recipient may also be high and lead to financial constraints for preventive health care or treatment among caregivers (35). This may be a particularly important factor in LMICs where high costs associated with treatment can even lead to catastrophic expenditures, especially in poor households (36).

As well as caregiving causing ill-health, it is also possible that a causal relationship exists in the opposite direction; that is, those who are ill are more likely to be caregivers. For instance, multimorbidity has a negative impact on labor market participation, and a Canadian study identified chronic physical conditions (eg, heart disease, arthritis, diabetes) and multimorbidity as risk factors for unemployment (37). Therefore, people with physical multimorbidity may be more available for informal caregiving activities (eg, personal care, household activities, supervision) than those without physical multimorbidity because they are more likely to be unemployed. The fact that the estimates remained statistically significant after adjustment for employment status in our study may mean that this explanation is unlikely, but there may be other reasons why an individual would be more likely to be at home or available for caregiving and thus, firm conclusions regarding reverse causality cannot be drawn from our cross-sectional study. Clearly, future longitudinal studies are warranted to understand temporal associations.

Interestingly, there was no significant association between informal caregiving and physical multimorbidity in the older adults in this study. Although the reason for this can only be speculated, it is possible that older adults with physical multimorbidity are too dependent to provide care for someone else or that basal levels of physical multimorbidity are already high in the older adult population. Alternatively, it is also possible that older people are less likely to be caregivers as older family members (eg, spouse) in need of informal care may have died. Future studies that investigate age differences in the association between caregiving and physical multimorbidity, ideally using longitudinal data, are warranted to understand the underlying mechanisms.

### Clinical Implications and Directions for Future Research

Regardless of whether informal caregiving and physical multimorbidity are causally related, our study highlights the fact that informal caregivers in LMICs are more likely to have worse physical health, and these study results are important when planning strategies to improve the health and well-being of informal caregivers to sustain this system. The fact that informal caregivers are more likely to have chronic physical conditions or physical multimorbidity is a particular concern as informal caregiving activities are often stressful, and stress may aggravate the symptoms of multiple chronic conditions (eg, pain, wheeze, functional limitation) (38), have a negative impact on treatment adherence (39), and increase mortality, especially among those with multimorbidity (40). Interventions reducing burden, improving support and favoring respite in caregivers may help prevent the onset of chronic physical conditions and improve the management of these conditions when they are already present (41). For example, a systematic review and meta-analysis of 32 randomized controlled trials showed that psychoeducation was associated with a significant decrease in global morbidities and perceived burden in family carers of people with psychosis (42). Another cross-sectional study further found in almost 600 participants that home nursing care had a significant and positive impact on self-rated health in caregivers aged 65 years or older (43). Finally, a study of 39 patients with dementia and their caregivers revealed that there was a positive relationship between institutional respite periods, total sleep time per night, total time in bed per night, and improvements in subjective sleep quality (44).

Treating patients with physical multimorbidity can be complex (eg, polypharmacy, simultaneous treatments) and requires a continuity of care with extended consultations to manage multiple conditions at once. However, it is possible that in many LMICs, treatment systems may lack integration of care or the capacity to provide this level of service quality (45). Policies that are most effective in the context of financial constraints should be established. Furthermore, further studies of longitudinal design are needed to gain a better understanding of the potential causality of the association between informal caregiving and physical multimorbidity, and of the potential mediators involved in this relationship. Finally, given that the prevalence of informal caregiving is also high in high-income countries (46), more research is required to corroborate the findings of the present study in these countries.

### Strengths and Limitations

The strengths of this study are the large number of participants and countries available for analysis, and the use of predominantly nationally representative data. However, there are several potential limitations that should be acknowledged. First, no information was available on the intensity or duration of informal caregiving, and there may be some differences in terms of the informal caregiving–physical multimorbidity relationship between high- and low-intensity caregiving. Relatedly, there was no information on the underlying disease of the care recipient. Some health conditions, such as urinary incontinence or Parkinson’s disease, are known to be more burdensome to the caregiver (47) and thus may have a more pronounced impact on caregiver health. Second, information on the medical conditions were based on self-report, and this may have introduced some biases. For example, arthritis, asthma, and diabetes mellitus were based solely on self-reported lifetime diagnosis, and there is a potential for underdiagnosis especially in resource-limited settings. Furthermore, tuberculosis diagnosis was based solely on typical symptoms of this condition, and thus, there is the potential for misclassification. Third, the definition of physical multimorbidity relied on the use of nine physical conditions only, and results may have differed if data on more conditions were available. Finally, this was a cross-sectional analysis, and it was therefore not possible to determine causality or temporality in the association between informal caregiving and multimorbidity.

### Conclusions

Informal caregivers in LMICs were more likely to have physical multimorbidity. Strategies to improve the physical health of caregivers in LMICs are necessary because the system of informal care provision cannot be sustained unless the caregivers are in good health, while many countries rely heavily on this system for the care of the older adults or people with disability.

## Supplementary Material

glaa017_suppl_Supplementary_Appendix

## Data Accessibility

The data sets supporting the conclusions of this article are available in the World Health Survey repository at http://www.who.int/healthinfo/survey/en/.

## References

[1] Dzau VJ, Jenkins JAC Creating a global roadmap for healthy longevity. J Gerontol A Biol Sci Med Sci. 2019;74(suppl 1):S4–S6. doi:10.1093/gerona/glz226 31690929

[2] World Health Organization. Aging and Health. https://www.who.int/news-room/fact-sheets/detail/ageing-and-health. Published 2018. Accessed 20 December, 2019.

[3] GBD 2017 Disease and Injury Incidence and Prevalence Collaborators. Global, regional, and national incidence, prevalence, and years lived with disability for 354 diseases and injuries for 195 countries and territories, 1990–2017: a systematic analysis for the Global Burden of Disease Study 2017. Lancet Lond Engl. 2018;392:1789–1858. doi:10.1016/S0140-6736(18)32279-7 PMC6227754 30496104

[4] Yao S-S, Cao G-Y, Han L, et al. Prevalence and patterns of multimorbidity in a nationally representative sample of older Chinese: results from CHARLS. J Gerontol A Biol Sci Med Sci. 2019. Advance online publication. doi:10.1093/gerona/glz185 31406983

[5] Broese van Groenou MI, De Boer A Providing informal care in a changing society. Eur J Ageing. 2016;13:271–279. doi:10.1007/s10433-016-0370-7 27610055 PMC4992501

[6] Thrush A, Hyder A The neglected burden of caregiving in low- and middle-income countries. Disabil Health J. 2014;7:262–272. doi: 10.1016/j.dhjo.2014.01.003 24947567

[7] Koyanagi A, DeVylder JE, Stubbs B, et al. Depression, sleep problems, and perceived stress among informal caregivers in 58 low-, middle-, and high-income countries: a cross-sectional analysis of community-based surveys. J Psychiatr Res. 2018;96:115–123. doi: 10.1016/j.jpsychires.2017.10.001 29031131

[8] Su P, Ding H, Zhang W, et al. The association of multimorbidity and disability in a community-based sample of elderly aged 80 or older in Shanghai, China. BMC Geriatr. 2016;16. doi:10.1186/s12877-016-0352-9 PMC5081877 27784269

[9] Lange-Maia BS, Karvonen-Gutierrez CA, Kazlauskaite R, et al. Impact of chronic medical condition development on longitudinal physical function from mid- to early late-life: the study of women’s health across the nation. J Gerontol A Biol Sci Med Sci. 2019. Advance online publication. doi:10.1093/gerona/glz243 PMC7302170 31732730

[10] Williams JS, Egede LE The association between multimorbidity and quality of life, health status and functional disability. Am J Med Sci. 2016;352:45–52. doi:10.1016/j.amjms.2016.03.004 27432034

[11] Jani BD, Hanlon P, Nicholl BI, et al. Relationship between multimorbidity, demographic factors and mortality: findings from the UK Biobank cohort. BMC Med. 2019;17:74. doi:10.1186/s12916-019-1305-x 30967141 PMC6456941

[12] Kim D Relationships between caregiving stress, depression, and self-esteem in family caregivers of adults with a disability. Occup Ther Int. 2017;2017:1686143. doi:10.1155/2017/1686143 29114184 PMC5664279

[13] Sacco LB, Leineweber C, Platts LG Informal care and sleep disturbance among caregivers in paid work: longitudinal analyses from a large community-based Swedish cohort study. Sleep. 2017;41. doi:10.1093/sleep/zsx198 PMC6018987 29228400

[14] Salgado-García FI, Zuber JK, Graney MJ, Nichols LO, Martindale-Adams JL, Andrasik F Smoking and smoking increase in caregivers of Alzheimer’s patients. Gerontologist. 2015;55:780–792. doi:10.1093/geront/gnt149 24371214 PMC4683364

[15] Fredman L, Bertrand RM, Martire LM, Hochberg M, Harris EL Leisure-time exercise and overall physical activity in older women caregivers and non-caregivers from the Caregiver-SOF Study. Prev Med. 2006;43:226–229. doi:10.1016/j.ypmed.2006.04.009 16737731

[16] Polenick CA, Leggett AN, Webster NJ, Han BH, Zarit SH, Piette JD Multiple chronic conditions in spousal caregivers of older adults with functional disability: associations with caregiving difficulties and gains. J Gerontol B Psychol Sci Soc Sci. 2017;160–172. doi:10.1093/geronb/gbx118 PMC6909432 29029293

[17] Frith E, Ramulu PY, Ashar B, Loprinzi PD Association of single and multiple medical conditions with work status among adults in the United States. J Lifestyle Med. 2019;9:15–26. doi:10.15280/jlm.2019.9.1.15 30918830 PMC6425902

[18] Payne CF, Pesando LM, Kohler H-P Private intergenerational transfers, family structure, and health in a sub-Saharan African context. Popul Dev Rev. 2019;45:41–80. doi:10.1111/padr.12225 32440034 PMC7241093

[19] Nuevo R, Chatterji S, Verdes E, Naidoo N, Arango C, Ayuso-Mateos JL The continuum of psychotic symptoms in the general population: a cross-national study. Schizophr Bull. 2012;38:475–485. doi:10.1093/schbul/sbq099 20841326 PMC3329982

[20] Freeman EE, Roy-Gagnon MH, Samson E, et al. The global burden of visual difficulty in low, middle, and high income countries. PLoS One. 2013;8:e63315. doi:10.1371/journal.pone.0063315 23675477 PMC3651198

[21] Smith L, Onwumere J, Craig T, McManus S, Bebbington P, Kuipers E Mental and physical illness in caregivers: results from an English national survey sample. Br J Psychiatry. 2014;205:197–203. doi:10.1192/bjp.bp.112.125369 25061119

[22] Vancampfort D, Koyanagi A, Ward PB, et al. Perceived stress and its relationship with chronic medical conditions and multimorbidity among 229,293 community-dwelling adults in 44 low- and middle-income countries. Am J Epidemiol. 2017;186:979–989. doi:10.1093/aje/kwx159 28637230

[23] Higgins JP, Thompson SG, Deeks JJ, Altman DG Measuring inconsistency in meta-analyses. BMJ. 2003;327:557–560. doi:10.1136/bmj.327.7414.557 12958120 PMC192859

[24] Darragh AR, Sommerich CM, Lavender SA, Tanner KJ, Vogel K, Campo M Musculoskeletal discomfort, physical demand, and caregiving activities in informal caregivers. J Appl Gerontol. 2015;34:734–760. doi:10.1177/0733464813496464 24652897 PMC3964150

[25] Punzi L, Galozzi P, Luisetto R, et al. Post-traumatic arthritis: overview on pathogenic mechanisms and role of inflammation. RMD Open. 2016;2:e000279. doi:10.1136/rmdopen-2016-000279 27651925 PMC5013366

[26] Capistrant BD, Moon JR, Glymour MM Spousal caregiving and incident hypertension. Am J Hypertens. 2012;25:437–443. doi:10.1038/ajh.2011.232 22189941 PMC3836043

[27] Wakabayashi C, Donato KM Does caregiving increase poverty among women in later life? Evidence from the health and retirement survey. J Health Soc Behav. 2006;47:258–274. doi:10.1177/002214650604700305 17066776

[28] Richardson S, Shaffer JA, Falzon L, Krupka D, Davidson KW, Edmondson D Meta-analysis of perceived stress and its association with incident coronary heart disease. Am J Cardiol. 2012;110:1711–1716. doi:10.1016/j.amjcard.2012.08.004 22975465 PMC3511594

[29] Gan Y, Gong Y, Tong X, et al. Depression and the risk of coronary heart disease: a meta-analysis of prospective cohort studies. BMC Psychiatry. 2014;14:371. doi:10.1186/s12888-014-0371-z 25540022 PMC4336481

[30] Lao XQ, Liu X, Deng HB, et al. Sleep quality, sleep duration, and the risk of coronary heart disease: a prospective cohort study with 60,586 adults. J Clin Sleep Med. 2018;14:109–117. doi:10.5664/jcsm.6894 29198294 PMC5734879

[31] Rod NH, Kristensen TS, Lange P, Prescott E, Diderichsen F Perceived stress and risk of adult-onset asthma and other atopic disorders: a longitudinal cohort study. Allergy. 2012;67:1408–1414. doi:10.1111/j.1398-9995.2012.02882.x 22943607

[32] Gallant MP, Connell CM Predictors of decreased self-care among spouse caregivers of older adults with dementing illnesses. J Aging Health. 1997;9:373–395. doi:10.1177/089826439700900306 10182399

[33] Pruchno RA, Potashnik SL Caregiving spouses. Physical and mental health in perspective. J Am Geriatr Soc. 1989;37:697–705. doi:10.1111/j.1532-5415.1989.tb02230.x 2754154

[34] Irfan B, Irfan O, Ansari A, Qidwai W, Nanji K Impact of caregiving on various aspects of the lives of caregivers. Cureus. 2017;9:e1213. doi:10.7759/cureus.1213 28589062 PMC5453737

[35] Ho A, Collins SR, Davis K, Doty MM A look at working-age caregivers’ roles, health concerns, and need for support. Issue Brief Commonw Fund. 2005;1–12. 16118908

[36] Leive A, Xu K Coping with out-of-pocket health payments: empirical evidence from 15 African countries. Bull World Health Organ. 2008;86:849–856. doi:10.2471/blt.07.049403 19030690 PMC2649544

[37] Smith P, Chen C, Mustard C, Bielecky A, Beaton D, Ibrahim S Examining the relationship between chronic conditions, multi-morbidity and labour market participation in Canada: 2000–2005. Ageing Soc. 2014;34:1730–1748. doi:10.1017/S0144686X13000457

[38] Oh YM, Kim YS, Yoo SH, Kim SK, Kim DS Association between stress and asthma symptoms: a population-based study. Respirology. 2004;9:363–368. doi:10.1111/j.1440-1843.2004.00609.x 15363009

[39] Aikens JE Prospective associations between emotional distress and poor outcomes in type 2 diabetes. Diabetes Care. 2012;35:2472–2478. doi:10.2337/dc12-0181 23033244 PMC3507577

[40] Prior A, Fenger-Grøn M, Larsen KK, et al. The association between perceived stress and mortality among people with multimorbidity: a prospective population-based cohort study. Am J Epidemiol. 2016;184:199–210. doi:10.1093/aje/kwv324 27407085

[41] Sörensen S, Pinquart M, Duberstein P How effective are interventions with caregivers? An updated meta-analysis. Gerontologist. 2002;42:356–372. doi:10.1093/geront/42.3.356 12040138

[42] Sin J, Gillard S, Spain D, Cornelius V, Chen T, Henderson C Effectiveness of psychoeducational interventions for family carers of people with psychosis: a systematic review and meta-analysis. Clin Psychol Rev. 2017;56:13–24. doi:10.1016/j.cpr.2017.05.002 28578249

[43] Chen MC, Kao CW, Chiu YL, et al. Effects of home-based long-term care services on caregiver health according to age. Health Qual Life Outcomes. 2017;15:208. doi:10.1186/s12955-017-0786-6 29061145 PMC5651602

[44] Lee D, Morgan K, Lindesay J Effect of institutional respite care on the sleep of people with dementia and their primary caregivers. J Am Geriatr Soc. 2007;55:252–258. doi:10.1111/j.1532-5415.2007.01036.x 17302663

[45] Navickas R, Petric VK, Feigl AB, Seychell M Multimorbidity: what do we know? What should we do? J Comorb. 2016;6:4–11. doi:10.15256/joc.2016.6.72 29090166 PMC5556462

[46] Verbakel E, Tamlagsrønning S, Winstone L, Fjær EL, Eikemo TA Informal care in Europe: findings from the European Social Survey (2014) special module on the social determinants of health. Eur J Public Health. 2017;27(suppl 1):90–95. doi:10.1093/eurpub/ckw229 28355645

[47] Brinda EM, Rajkumar AP, Enemark U, Attermann J, Jacob KS Cost and burden of informal caregiving of dependent older people in a rural Indian community. BMC Health Serv Res. 2014;14:207. doi:10.1186/1472-6963-14-207 24886051 PMC4022434

