# Supplementary material for: Informal Caregiving, Chronic Physical Conditions, and Physical Multimorbidity in 48 Low- and Middle-Income Countries
Source: J Gerontol A Biol Sci Med Sci. 2020 Jan 16;75(8):1572–8. doi: 10.1093/gerona/glaa017 (PMC7357583; doi:10.1093/gerona/glaa017)
Supplement: glaa017_suppl_Supplementary_Appendix [file glaa017_suppl_supplementary_appendix.docx]

**Appendix**

| **eTable 1** Sample size by country | | | |
| --- | --- | --- | --- |
| Country | N | Country | N |
| Bangladesh | 5,942 | Mali | 4,886 |
| Bosnia Herzegovina | 1,031 | Mauritania | 3,902 |
| Brazil | 5,000 | Mauritius | 3,968 |
| Burkina Faso | 4,948 | Mexico | 38,746 |
| Chad | 4,870 | Morocco | 5,000 |
| China | 3,994 | Myanmar | 6,045 |
| Comoros | 1,836 | Namibia | 4,379 |
| Croatia | 993 | Nepal | 8,820 |
| Czech Republic | 949 | Pakistan | 6,501 |
| Dominican Republic | 5,027 | Paraguay | 5,288 |
| Ecuador | 5,675 | Philippines | 10,083 |
| Estonia | 1,020 | Republic of Congo | 3,075 |
| Ethiopia | 5,089 | Russia | 4,427 |
| Georgia | 2,950 | Senegal | 3,461 |
| Ghana | 4,165 | Slovakia | 2,535 |
| Hungary | 1,419 | South Africa | 2,629 |
| India | 10,687 | Sri Lanka | 6,805 |
| Ivory Coast | 3,251 | Swaziland | 3,117 |
| Kazakhstan | 4,499 | Tunisia | 5,202 |
| Kenya | 4,640 | Ukraine | 2,860 |
| Laos | 4,988 | Uruguay | 2,996 |
| Latvia | 929 | Vietnam | 4,174 |
| Malawi | 5,551 | Zambia | 4,165 |
| Malaysia | 6,145 | Zimbabwe | 4,290 |
